# Within‐community variation of interspecific divergence patterns in passerine gut microbiota

**DOI:** 10.1002/ece3.9071

**Published:** 2022-07-04

**Authors:** Jan Kubovčiak, Lucie Schmiedová, Tomáš Albrecht, Martin Těšický, Oldřich Tomášek, Tereza Kauzálová, Jakub Kreisinger

**Affiliations:** ^1^ Department of Zoology, Faculty of Science Charles University Prague Czech Republic; ^2^ Institute of Vertebrate Biology Czech Academy of Sciences Brno Czech Republic

**Keywords:** 16S rRNA, co‐divergence, gut microbiota, metabarcoding, passerines

## Abstract

Gut microbiota (GM) often exhibit variation between different host species and co‐divergence with hosts' phylogeny. Identifying these patterns is a key for understanding the mechanisms that shaped symbiosis between GM and its hosts. Therefore, both GM‐host species specificity and GM‐host co‐divergence have been investigated by numerous studies. However, most of them neglected a possibility that different groups of bacteria within GM can vary in the tightness of their association with the host. Consequently, unlike most of these studies, we aimed to directly address how the strength of GM‐host species specificity and GM‐host co‐divergence vary across different GM clades. We decomposed GM communities of 52 passerine species (394 individuals), characterized by 16S rRNA amplicon sequence variant (ASV) profiles, into monophyletic Binned Taxonomic units (BTUs). Subsequently, we analyzed strength of host species specificity and correlation with host phylogeny separately for resulting BTUs. We found that most BTUs exhibited significant host‐species specificity in their composition. Notably, BTUs exhibiting high host‐species specificity comprised bacterial taxa known to impact host's physiology and immune system. However, BTUs rarely displayed significant co‐divergence with host phylogeny, suggesting that passerine GM evolution is not shaped primarily through a shared evolutionary history between the host and its gut microbes.

## Graphical abstract

Here, we decomposed gut microbiota communities of 52 passerine species (394 individuals), characterized by 16S rRNA amplicon sequence variant profiles, into monophyletic Binned Taxonomic units (BTUs). Subsequently, we analysed strength of host species specificity and co‐divergence with host phylogeny separately for resulting BTUs. We found that most BTUs exhibited significant host‐species specificity in their composition. However, BTUs rarely displayed significant co‐divergence with host phylogeny, suggesting that passerine GM evolution is not shaped primarily through a shared evolutionary history between the host and its gut microbes.

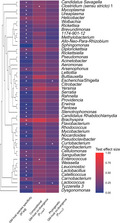

## 1 INTRODUCTION

Multicellular eukaryotic lifeforms are inevitably exposed to interactions with microorganisms, including bacteria, throughout their phylogeny and ontogeny (McFall‐Ngai et al., 2013). In animals, the quantity of associated bacterial cells represents a significant fraction of all cells forming the host's body (Sender et al., 2016). A similar situation applies for the total gene count of the bacterial community, which outnumbers the host's gene count by a factor of approx. 100 (Qin et al., 2010). The most abundant and functionally rich host‐associated bacterial community is found in the gut (gut microbiota, GM) (Marietta et al., 2015; Suau et al., 1999). As the gut wall represents one of the largest body surfaces in total area, it is broadly assumed that the GM possesses great potential to influence the host's physiology, immune system, or cognitive function (Bäckhed et al., 2005; Eckburg et al., 2005; Hedblom et al., 2018; Heijtz et al., 2011). Empowered by increased availability of high‐throughput sequencing, a plethora of findings over past decades have shown GM to be a crucial factor affecting host fitness (Bordenstein & Theis, 2015; Brucker & Bordenstein, 2013; Hooper et al., 2012; Sharon et al., 2010).

During both early life and adulthood, individuals are continuously colonized by bacteria of different origins. The most obvious GM sources are pools of environmental bacteria present in the host's physical surroundings (Sullam et al., 2012). Alternatively, GM can be transferred via social contacts with conspecifics (Colston & Jackson, 2016). At the same time, animal hosts are capable of regulating microbiota by various means, for example, by immune system utilities, making host genetic background an important factor shaping GM diversity (Marietta et al., 2015; Spor et al., 2011). This commonly results in variation in GM composition among different host species, that is, GM‐host species specificity.

It has been suggested that a host benefits from harboring a taxonomically and functionally stable GM community, and conserving such an association (Bäckhed et al., 2005; Knutie et al., 2017; Sanders et al., 2014), as it leads to a reduction in mutually harmful interactions and the emergence of commensal/mutualistic associations (Parker et al., 2017). At the proximal level, such a tight and stable association can be mediated either by the stable transfer of microbes from the environment or by trans‐generational transfer among members of a given host species. A stable host versus microbiota association, persisting over multiple generations and speciation events, often results in co‐divergence between hosts' phylogeny and their GM (Bordenstein & Theis, 2015). However, co‐divergence can also arise as an epiphenomenon of host traits that are linked to its phylogeny and that selectively filter for environmental microbes. GM‐host co‐divergence has been demonstrated in mammals, where host phylogeny, along with diet, significantly influences GM composition and diversity (Davenport, 2016; Ley et al., 2008; Moeller et al., 2016). Persuasive evidence of trans‐generation transfer and co‐divergence with hosts comes from obligatory bacterial symbionts of some insect groups (Weiss et al., 2012). These symbionts are unable to persist outside their hosts and some insect species have even developed specialized organs to harbor such microbes (Baumann, 2005). Symbiotic microbes are suspected of playing a direct role in speciation processes, such as assortative mating and fitness decrease in interspecific hybrids due to disruption of host versus microbiota co‐adaptations (Brucker & Bordenstein, 2013). At the same time, however, it is important to denote that co‐divergence does not necessarily imply co‐evolution between host and its GM. Although many GM bacteria evolved adaptation to specific hosts that enables them to follow its phylogeny, well‐supported cases when host developed specific adaptations to specific bacterial symbiont are rather unique (Douglas & Werren, 2016; Moran & Sloan, 2015).

One of the potential bottlenecks associated with research on GM variation arises from the complexity of GM structure. Insights into GM variation are usually gained through analyses at the beta diversity level of all detected bacteria, that is, the “whole GM community” (Bodawatta et al., 2021; Brooks et al., 2016; Capunitan et al., 2020; Hird et al., 2015; Trevelline et al., 2020). However, such an approach may prevent a more realistic insight into the complexity of host versus GM interactions. Indeed, different phylogenetic clades of GM bacteria may exhibit distinct modes of interactions with their hosts (Davenport et al., 2015; Douglas & Werren, 2016; Youngblut et al., 2019). To overcome potential problems associated with the fact that different GM components exhibit a heterogeneous response to a tested variable (e.g., host phylogeny and diet), some previous studies have decomposed GM into subsets usually defined by the taxonomic identity of the bacteria they contain. Then, for each of these subsets, the strength of association between the variable tested and the variation in composition within each of the GM the subsets was examined. This allowed the identification of GM subsets that respond strongly (or weakly) to the tested variable (Dewar et al., 2013; Houtz et al., 2021; Youngblut et al., 2019). We believe that similar approaches can provide novel insight into patterns of GM diversity and will be particularly valuable for research on GM‐host co‐divergence and specificity.

In our current contribution, we focused on GM vs host species co‐divergence in passerine birds that represent a promising model system for such research. Being evolutionary and ecologically the most diversified avian group, they span a wide spectrum of habitats, resulting in differing demands on host‐microbiome interactions. Despite recent radiation (approx. 22 million years ago), their phylogeny is mostly well resolved (Jetz et al., 2012; Moyle et al., 2016) and a plethora of life history traits have been documented, providing a strong background for the proposed analysis. So far, there is an agreement that host species identity is a considerable factor shaping GM of birds, but to lesser extent than in other vertebrate clades, such as mammals (Song et al., 2020; Trevelline et al., 2020; Youngblut et al., 2019, but see Cho & Lee, 2020; Lewis et al., 2017). Similarly, host ecology seems to play less important role in birds compared to mammals (Song et al., 2020). Such peculiarity may have arisen due to adaptations of avian digestive system to flight, constraining relative size of individual internals (Bodawatta et al., 2021). Specifically, caeca and other parts of the digestive system are typically reduced in passerines, resulting in lesser potential for physiologically significant and stable host–microbiota interactions (Bodawatta et al., 2022; Caviedes‐Vidal et al., 2007). Major differences between GM of mammals and birds are found also in representation of major bacterial phyla. There is higher proportion of *Proteobacteria* and *Actinobacteria* in GM of birds (Capunitan et al., 2020; Grond et al., 2018) including passerines (Kropáčková et al., 2017), while *Bacteroidetes* and *Firmicutes* typically dominate mammalian GM (Ley et al., 2008).

In our previous contribution, we found that passerine GM composition exhibited only mild interspecific differences that were, to a large extent, correlated with hosts phylogeny but, surprisingly, not with their ecology (Kropáčková et al., 2017). However, the basis of these patterns has yet to be uncovered. Specifically, the observed interspecific differences in GM, as well as GM vs. host phylogeny correlations, could be driven by just a few bacterial clades, while the rest of the GM community has no dependence on host species. Here, we aimed to address how different bacterial clades contribute to interspecific variance of the host and co‐divergence at the whole GM community level. To achieve this goal, we analyzed passerine GM profiles in three subsequent steps: First we applied precise clustering‐free approach (Callahan et al., 2016) to identify distinct 16S rRNA haplotypes (hereafter ASVs, i.e., Amplicon Sequence Variants) and to quantify their relative abundances in each sample. Next, motivated by previous studies that have performed analyses separately for individual subgroups of the entire GM (e.g., Dewar et al., 2013; Houtz et al., 2021; Youngblut et al., 2019), we decomposed the entire GM community into presumably monophyletic ASVs subunits and hereafter referred to these as Binned Taxonomic Units (BTUs). We considered two approaches of BTU definition, (1) the “reference‐based,” where we binned individual ASVs according to their genus‐level taxonomic assignment and (2) the “reference‐free,” where we binned ASVs based on their 16S rRNA sequence similarity (see Methods section for more details). Finally, we quantified the strength of GM‐host species specificity and GM‐host species co‐divergence independently for each of these BTUs. This approach allows us to draw conclusions at the level of particular groups of bacteria (represented by individual BTUs), which is useful for identifying BTUs that drive host species specificity or co‐divergence at the level of the entire GM.

## 2 MATERIAL AND METHODS

### 2.1 Field sampling and microbiota genotyping

In this study, we analyzed data on passerine GM that were already published in Kropáčková et al. (2017). We collected 480 fecal samples from 57 species during the breeding season (April–July 2014) across various sites in the Czech Republic. But due to unsuccessful polymerase chain reaction (PCR) or low number of reads (detailed below), only 394 GM profiles from different individuals covering 52 passerine species (51% of all passerine species breeding in the Czech Republic) were included in the final dataset and subsequent analyses (see Table S1). Birds were caught using mist nets and placed in a single‐use disposable paper bag for approx. 5–10 min. Fecal samples were harvested from the bag using sterile microbiological swabs (minitip FLOQSwabs, Copan, Italy) and transferred to sterile cryotubes (Simport, Canada) which were then filled with an in‐house prepared DNA/RNA stabilizing solution. The samples were then cooled to −80°C within 5 days and stored until further analysis.

Fecal metagenomic DNA was extracted in a laminar flow cabinet using the PowerSoil DNA isolation kit (MO BIO Laboratories Inc., USA). Following the recommendations of Klindworth et al. (2013), primers covering the V3–V4 variable region of bacterial 16S rRNA were used during the PCR step. Both forward and reverse primers were tagged with 10‐bp barcodes for subsequent sample demultiplexing during bioinformatic processing. The samples were then pooled at equimolar concentration and run on 1.5% agarose gel, with bands of appropriate size excised from the gel and purified using the High Pure PCR product Purification Kit (Roche, Switzerland), according to the manufacturer's instructions. Sequencing adaptors were ligated using TruSeq nano DNA library preparation kits (Illumina, USA) and the resulting amplicon libraries sequenced in a single MiSeq run (Illumina, USA) using v3 chemistry and 2 × 300 nt paired‐end configuration. For more detailed protocols on field sampling, wet lab procedures and sequencing methods, see Kropáčková et al. (2017).

### 2.2 Computational procedures

**FIGURE 1:**
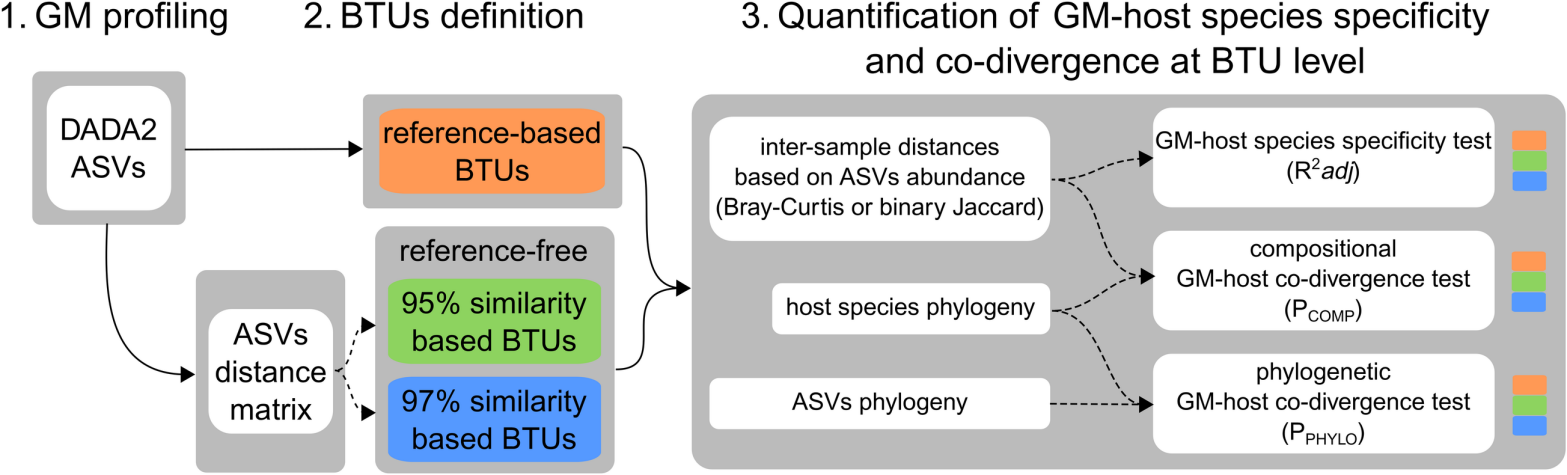
Analytical approach used in our study. 1. GM profiling: Raw paired‐end reads were merged and sequencing errors were corrected by DADA2 to obtain amplicon sequence variants (ASVs). 2. BTUs definition: ASVs were grouped to BTUs using three methods in parallel, namely a) reference based taxonomic assignment of ASVs to genus level and reference free methods defining BTUs sharing at least b) 95% or c) 97% ASVs sequence similarity, based on distance matrix. 3. Quantification of GM‐host species specificity and co‐divergence at the BTU level: Several tests for different aspects of GM diversity were conducted on ASVs distribution within individual BTUs, namely a) GM‐host species specificity test based on permutational MANOVA of inter‐sample GM composition distances (Bray–Curtis and Jaccard), b) compositional GM‐host co‐divergence test based on PACo analyses of inter‐sample GM composition distances and host phylogeny, c) phylogenetical GM‐host co‐divergence test based on PACo analyses of ASVs phylogeny within individual BTUs and hosts phylogeny

Our aim was to decompose the GM community to putatively monophyletic bacterial subunits (hereafter BTU, i.e., Binned Taxonomic Unit), and subsequently to quantify the strength of GM versus host species co‐divergence for each of these subunits. To address these objectives, computational procedures comprised three major stages: 1. GM profiling that constituted identification and quantification of ASVs, 2. BTUs definition, and 3. quantification of GM‐host species specificity and co‐divergence at the BTUs level based on ASVs variation within each BTU (for an overview of the computational procedure design, see Figure 1).

#### 2.2.1 GM profiling

We used the DADA2 program (Callahan et al., 2016) for read quality filtering (per paired‐end read expected error rate <1), read pair merging and ASV identification. This approach outperforms methods relying on similarity‐based clustering into Operational Taxonomic Units (OTUs) through its use of a real variant inference algorithm. Unlike clustering approaches, DADA2 rather corrects sequencing errors, resulting in the formation of more reliable biological units (ASVs). Importantly, DADA2 can detect variation up to single base pair differences, which far exceeds the resolution provided by standard clustering algorithms. USEARCH (Edgar, 2010), alongside the gold.fna database (available at http://sourceforge.net/projects/microbiomeutil), was subsequently used for the detection and elimination of chimeric ASVs. The resulting ASV abundance matrices, along with sample data, were then merged into PHYLOSEQ‐class objects in R for further analysis (McMurdie & Holmes, 2013; R Core Team, 2015).

ASV taxonomy was assigned using the RDP classifier (Wang et al., 2007) and the Silva Reference Database version 123 (Quast et al., 2013), using a default minimum bootstrap confidence value of 50 for assigning up to a given taxonomic level. All ASVs unassigned to at least phylum level, or those assigned as Chloroplasts, Archea or mitochondria, were discarded from further analysis, as were samples with <1000 reads in total. The final filtered dataset included samples for 394 individuals from 52 species (median = 8 samples per species), represented by 2,353,438 high‐quality reads assigned to 10,583 ASVs. Average number of reads per sample was 5973.2 ± 242.9 SE.

#### 2.2.2 Reference‐based and reference‐free BTUs definition

We applied two distinct approaches for defining putatively monophyletic BTUs. In the case of reference‐based approach, we grouped ASVs based on genus‐level assignment, resulting in 70.7% of ASVs being assigned to 862 known genera (i.e., reference‐based BTUs). However, bacterial taxonomy undergoes rapid evolution and thus there are inconsistencies between different taxonomic databases (Balvočiūtė & Huson, 2017). Also, ASVs with no genus‐level assignment cannot be used. Moreover, there is pronounced variation in sequence similarity between genus‐level bins.

To circumvent these issues, we also applied reference‐free BTUs definition based on ASVs clustering into putatively monophyletic subunits according to their 16S rRNA marker sequence similarity. While there is no universal 16S rRNA similarity threshold for bacterial clade delimitation, a sequence similarity of 97–98.65% has been suggested as an approximate threshold delimiting bacterial species (Kim et al., 2014). Hence, we grouped ASVs exhibiting >97% sequence similarity in order to acquire biologically meaningful clusters (i.e., 97% similarity‐based BTUs, see Figure 1). Finally, ASVs were clustered using a 95% similarity threshold (i.e., 95% similarity‐based BTUs) to explore GM diversity patterns at a higher taxonomic level corresponding approximately to bacterial genera (Yarza et al., 2014, but see Beye et al., 2018; Rossi‐Tamisier et al., 2015).

In order to cluster ASVs in the reference‐free way, we used the approach proposed in the R package DECIPHER (Wright, 2015) and VSEARCH (Rognes et al., 2016) clustering, that is widely used for 16S rRNA amplicon sequencing data. Both these methods showed comparable results, though DECIPHER resulted in less taxonomically ambiguous and more significant BTUs (Table S4). Moreover, DECIPHER, but not VSEARCH (and other traditional greedy clustering algorithms as well), implicitly accounts for phylogenetic relatedness of 16 s rRNA sequences. Therefore, we based our results on DECIPHER clustering. In the case of DECIPHER approach, ASV marker sequences were aligned using the ALIGNSEQS function. Next, a distance matrix based on Hamming distances between aligned ASVs was computed assuming the Jukes–Cantor substitution model. Finally, ASVs were clustered into monophyletic units based on the distance matrix using the IDCLUSTERS function, using the complete‐linkage criterion. In this way, all ASVs in a given BTU shared at least the pre‐defined sequence similarity value. We identified total of 2664 95% similarity‐based BTUs and 3932 97% similarity‐based BTUs. For all subsequent analyses, we only selected BTUs represented by at least 5000 reads and 10 bacterial ASVs, BTUs not meeting these criteria being omitted in order to avoid spurious results. These thresholds were defined based on a pilot analysis showing that variation in the strength of interspecific signal and co‐divergence was inordinate in the case of low diversity (consisting of <10 ASVs) and/or rare BTUs (< 5000 reads). The filtering criteria applied resulted in selection of 49 reference‐based BTUs (median = 40 ASVs), representing 71% of all reads; 58 95% similarity‐based BTUs BTUs (median = 28.5 ASVs), representing 70% of all reads; and 57 97% similarity‐based BTUs (median = 20 ASVs), representing 57% of all reads.

#### 2.2.3 Quantification of GM‐host species specificity and co‐divergence at the BTUs level

We calculated dissimilarities in ASVs composition among samples separately for each BTU using two methods, Bray–Curtis and the binary version of Jaccard distance. The Bray–Curtis method quantifies the compositional dissimilarity between samples while considering the quantity of ASVs in individual samples. Binary version of Jaccard distance (Jaccard method applied to presence or absence scaled ASV abundances), on the other hand, only evaluates ASV presence and absence, which makes it more sensitive to the appearance of rare bacterial groups. ASV abundance was transformed to the proportion of total library sizes for each sample prior to dissimilarity computation. In parallel with the Jaccard and Bray–Curtis dissimilarities, we also performed GM‐host species specificity analyses using weighted and unweighted UniFrac dissimilarities that account for bacterial phylogeny by downweighting the divergence caused by related bacterial ASVs. The strength of GM‐host species specificity analyses at the BTU level for UniFrac dissimilarities was comparable to those for their phylogenetically naive counterparts (Jaccard and Bray–Curtis) and thus we do not report these results further. On the other hand, we did not use UniFrac for co‐divergence analyses because the reduction in importance of closely related ASVs makes interpretation of the corresponding results problematic, except in certain cases (e.g., Kropáčková et al., 2017; Sanders et al., 2014).

To assess the strength of GM‐host species specificity for each BTU, PERMANOVA (i.e., Permutational Multivariate Analysis of Variance for distance matrices; R package VEGAN, Oksanen et al., 2016) was applied. To test whether phylogenetic divergence between species was correlated with community divergence at the level of each BTU, we used PACo (Procrustean Approach to Cophylogeny) analysis (Balbuena et al., 2013) that was originally developed for the assessment of host versus parasite cophylogeny.

In the case of PERMANOVA analysis, we included host species identity as an explanatory variable and the dissimilarity matrix for each BTU (scaled using both distance methods) as a response. We did not include host species life histories traits or other ecological features as explanatory variable, as they have little effect on GM in this dataset (Kropáčková et al., 2017) and PERMANOVA is not suitable for these types of analyses, as it cannot account for the dependence of ecological traits on host phylogeny. Statistical significance was assessed using 10,000 permutation rounds. To allow direct comparisons between BTUs, adjusted *R* ^2^ coefficients (*R* ^2^adj), that is, the proportion of variance explained by species identity corrected for different degrees of freedom and number of observations in species/individuals hosting a given BTUs, were calculated for each BTU analyzed (Legendre et al., 2011). In effect, therefore, *R* ^2^adj represents the strength of GM‐host species specificity of ASVs within each BTU.

As an explanatory variable for PACo analysis, we used cophenetic distances between host species that were extracted from consensual phylogeny calculated based on a set of 1000 Bayesian trees with Hackett backbone (obtained from http://birdtree.org/; Jetz et al., 2012). In order to quantify compositional GM‐host co‐divergence strength (P_COMP_), a PACo response was considered as either the Bray–Curtis or Jaccard GM dissimilarities for ASVs within individual BTUs. Though this approach has been commonly used in many previous studies on host versus GM co‐divergence (Capunitan et al., 2020; Hird et al., 2015; Song et al., 2020; Youngblut et al., 2019), it completely ignores bacterial phylogeny, that can be crucial in this particular context. Therefore, in parallel to traditionally used community dissimilarities associated with P_COMP_, we also analyzed phylogenetic GM‐host co‐divergence (P_PHYLO_), which is based on the genetic distance among ASVs within a given BTU. Genetic distances were calculated with DIST.DNA function from R package APE (Paradis et al., 2004) after their marker sequence alignment (using the same algorithm described above). A suitable substitution model for the distance calculation was selected separately for each BTU based on the lowest AICc using the MODELTEST function from the R package PHANGORN (Schliep, 2011). Both explanatory (host species phylogeny) and response (phylogeny or diversity of ASVs) distance matrices were scaled using Principal Coordinate Analysis (PCoA) prior to PACo fitting and the resulting PCoA score matrices were used as PACo inputs. As a result, we obtained Procrustes correlation coefficients expressing strength of co‐divergence in each BTU. Significance testing was based on a comparison of observed versus permuted Procrustes sum of squares (*n* = 10,000 permutations). To account for the fact that we typically analyzed multiple samples for each species, species identity was reshuffled across blocks of species‐specific samples during the permutation routine, as described in Kropáčková et al. (2017).

In addition, we also used the Mantel test to quantify the correlation of host phylogeny and GM composition divergence at the level of individual BTUs. We compared Bray–Curtis GM dissimilarities based on GM profiles averaged per host species against host cophenetic distances (the same as in PACo analysis). We found out that Mantel test exhibited uniformly insignificant results for all BTUs, while the respective effect sizes (Mantel's *r*) correlated tightly with the PACo correlation coefficients. Because of the known concerns with using Mantel tests in different contexts, including phylogenetic comparative methods (Guillot & Rousset, 2013; Harmon & Glor, 2010), we decided to base our analyses on the PACo approach.

For both PERMANOVA and PACo, False Discovery Rates (FDR; Benjamini & Hochberg, 1995) were applied in order to reduce false positives due to multiple testing.

Finally, we also analyzed overall GM‐host species specificity (represented by *R* ^2^) and overall compositional GM‐host co‐divergence (P_COMP_) for the full dataset unpartitioned to BTUs (the non‐binned raw ASV data, i.e., whole GM community analysis) and compared these results with patterns obtained for individual BTUs.

## 3 RESULTS

### 3.1 Whole GM community analysis summary

Using the full dataset unpartitioned to BTUs, we observed significant GM‐host species specificity (*R* ^2^ = 0.21, *p* < .001 for Bray–Curtis and *R* ^2^ = 0.18, *p* < .001 for Jaccard) and significant compositional GM‐host co‐divergence (P_COMP_ = 0.35, *p* = .015 for Bray–Curtis and P_COMP_ = 0.34, *p* = .018 for Jaccard).

### 3.2 Variation between BTU definition methods and co‐divergence metrics

Analyses of GM‐host species specificity and compositional GM‐host co‐divergence running on Bray–Curtis and Jaccard distances provided highly congruent result (Kendall's *τ* > 0.81, *p* < .001 for all comparisons) irrespective of BTU definition method (i.e., reference‐based BTUs, 97% or 95% similarity‐based BTUs; Figure S1). As such, further discussion is restricted to analysis based on Jaccard distance only.

**FIGURE 2:**
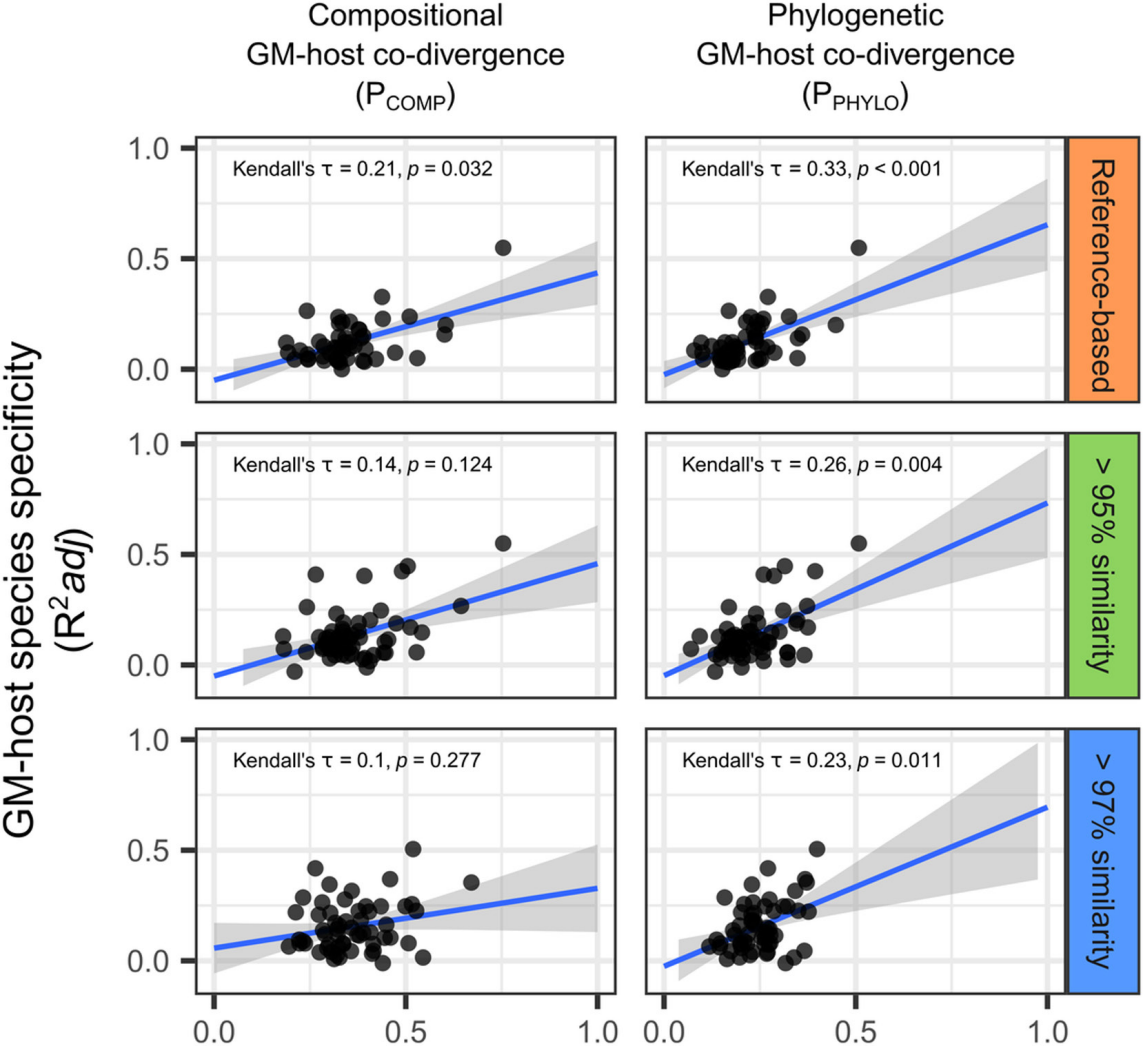
Correlations of test effect sizes in examined BTUs. Correlation of GM‐host species specificity versus compositional and phylogenetic GM‐host co‐divergence among BTUs (represented by points). Only a subset BTUs passing filters for testing are shown. Kendall's tau was used as the correlation coefficient. The blue line and shaded area correspond to predictions of linear regression and 95% confidence intervals, respectively. Only results for Jaccard distances are shown

In the case of reference‐based BTUs, GM‐host species specificity (*R* ^2^adj) exhibited a significant correlation, though of small effect size, with compositional GM‐host co‐divergence (*P*_COMP_) (Kendall's *τ* = 0.21, *p* = .03). This was not true for analyses on 95% similarity‐based BTUs (Kendall's *τ* = 0.14, *p* = .12) and 97% similarity‐based BTUs (Kendall's *τ* = 0.1, *p* = .28; Figure 2). Furthermore, both GM‐host species specificity and compositional GM‐host co‐divergence correlated with phylogenetic GM‐host co‐divergence (P_PHYLO_) among individual BTUs for all BTU definition methods (Kendall's *τ* > 0.23, *p* < .011; Figure 2).

### 3.3 Reference‐based BTUs analysis

**FIGURE 3:**
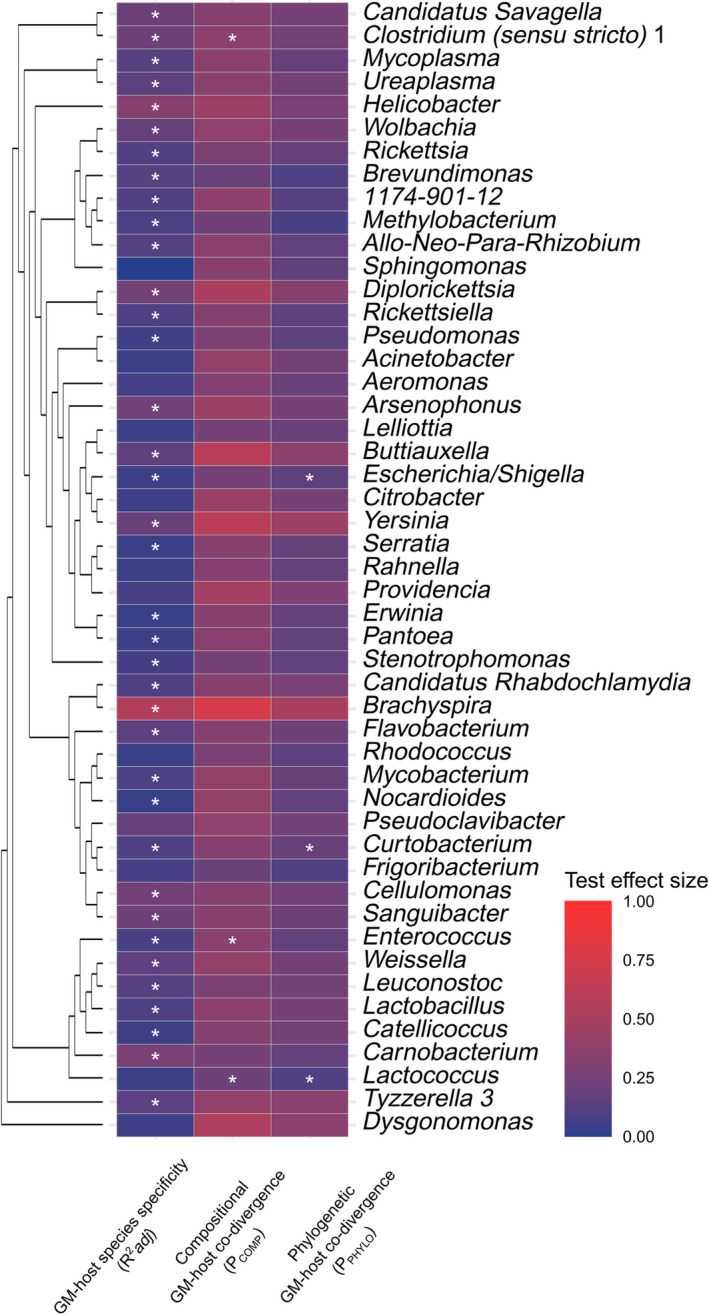
Heatmap of measured effect sizes in tested reference‐based BTUs. GM‐host species specificity (*R* ^2^adj) and compositional GM‐host co‐divergence (P_COMP_) are based on Jaccard distances. * indicates significant result (FDR < 0.05), the dendrogram on the left represents phylogeny of the bacterial genera, that is, a phylogenetic tree based on 16S rRNA sequences extracted from GreenGenes database (97% OTUs tree version 12_10) formatted for the purpose of QIIME1)

**FIGURE 4:**
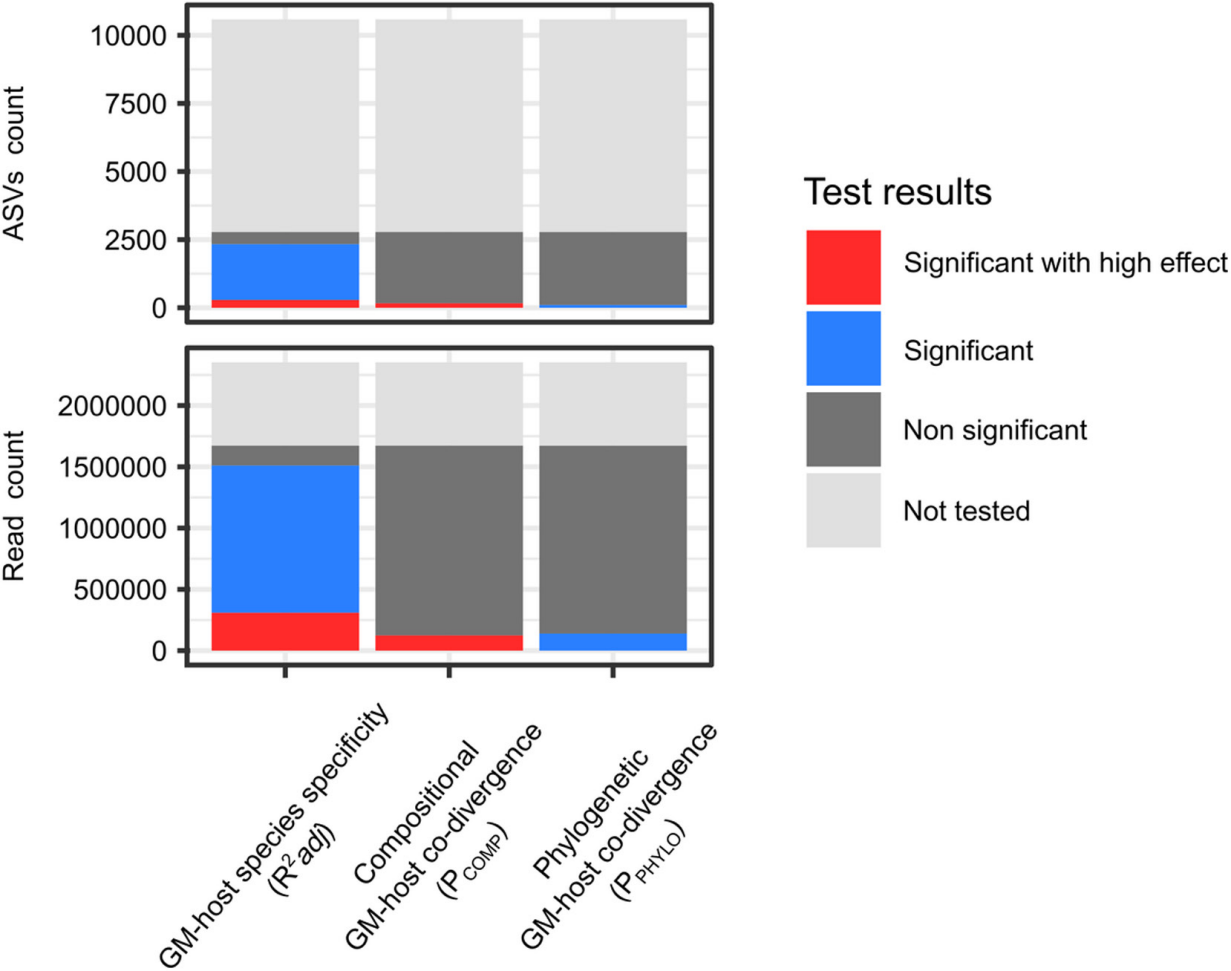
Whole GM community classified according to outcomes of BTU‐level analyses. The figure depicts GM fractions that were not included in BTU‐level analyses, where corresponding BTU level analysis was nonsignificant, significant or significant with high effect‐size (*R* ^2^adj/P_COMP_/P_PHYLO_ > 0.2). GM fractions are expressed as (a) total number of ASVs or (b) proportion of all reads. Note that, while modest number of ASVs were actually tested, they represented the majority of all reads in the dataset

GM‐host species specificity was significant (FDR < 0.05) for the majority (75%) of the 49 reference‐based BTUs examined, though overall effect sizes were moderate (*R* ^2^adj < 0.2) in most cases (Figure 3, Tables S2 and S3). Nevertheless, a subset of 10 reference‐based BTUs, represented by 13% of all reads and 3% of all ASVs (Figure 4), exhibited a high degree of GM‐host species specificity (*R* ^2^adj ≥0.2, FDR <0.05; Table 1). The strength of GM‐host species specificity for all ASVs included in these 10 BTUs (286 ASVs in total) was markedly higher (*R* ^2^adj = 0.131, *p* = .001) than GM‐host species specificity for all other ASVs not included in this subset (10,297 ASVs in total; *R* ^2^adj = 0.053). These results were supported by permutation analysis, which showed that strength of GM‐host species specificity for the 286 ASVs whose incidence varied strongly between host species was significantly higher than GM‐host species specificity for 286 ASVs randomly picked from the full dataset (*n* = 9999 permutations, permutation‐based *p* = .0002). The most abundant BTU exhibiting significant GM‐host species specificity consisted of ASVs from the genera *Candidatus Arthromitus*. ASVs found in these BTUs are in fact members of the genera *Candidatus Savagella,* which had not been defined in the reference database by the time of its release.

**TABLE 1:** Results of GM‐host species specificity and GM‐host co‐divergence tests for selected BTUs

Genus assignment	BTU label	Hosting individuals count	Hosting species count	ASVcount	ASVs abundance (×1000)	GM‐hostspeciesspecificity (*R* ^2^adj)	Compositional GM‐host co‐divergence (P_COMP_)	Phylogenetical GM‐host o‐divergence (P_PHYLO_)
Reference‐based BTUs								
*Candidatus Savagella*	–	192	44	40	108.5	0.21*	0.33	0.25
*Escherichia/Shigella*	–	226	47	59	107.3	0.05*	0.25	0.15*
*Cellulomonas*	–	184	48	21	59.2	0.24*	0.32	0.23
*Enterococcus*	–	233	52	91	53	0.08*	0.34*	0.16
*Clostridium (*sensu stricto*) 1*	–	195	45	56	52.3	0.22*	0.35	0.24
*Helicobacter*	–	97	38	20	21.9	0.33*	0.44	0.27
*Lactococcus*	–	185	47	16	18.7	0.04	0.21*	0.10*
*Carnobacterium*	–	143	44	14	17.4	0.26*	0.24	0.17
*Arsenophonus*	–	68	34	23	13.8	0.23*	0.44	0.26
*Diplorickettsia*	–	65	33	26	13	0.24*	0.51	0.33
*Curtobacterium*	–	142	46	27	13	0.10*	0.32	0.19*
*Yersinia*	–	50	27	51	9.9	0.20*	0.6	0.45
*Sanguibacter*	–	101	39	21	7.5	0.21*	0.33	0.21
*Brachyspira*	–	30	15	14	5.4	0.55*	0.75	0.51
95% similarity based BTUs								
*Candidatus Savagella*	BTU_478	189	44	20	107.8	0.23*	0.32	0.24
*Escherichia/Shigella*	BTU_47	224	47	50	107.1	0.06*	0.24	0.15*
*Clostridium (*sensu stricto*) 1*	BTU_549	129	38	14	37.9	0.41*	0.27	0.26
*Arsenophonus*	BTU_100	64	32	20	13.2	0.25*	0.44	0.31
*Clostridium (*sensu stricto*) 1*	BTU_538	48	28	15	9.9	0.40*	0.39	0.29
*Helicobacter*	BTU_528	48	24	10	7.8	0.42*	0.49	0.39
*Brachyspira*	BTU_573	30	15	14	5.4	0.55*	0.75	0.51
97% similarity based BTUs								
*Rickettsiella*	BTU_269	161	45	14	69.6	0.22*	0.21	0.20
*Candidatus Savagella*	BTU_643	122	38	12	45.1	0.32*	0.36	0.34
*Mycoplasma*	BTU_291	86	27	20	43.2	0.28*	0.34	0.28
*Clostridium (*sensu stricto*) 1*	BTU_735	129	38	12	37.7	0.42*	0.26	0.27
unassigned	BTU_280	69	34	10	34.1	0.35*	0.30	0.23
*Ureaplasma*	BTU_220	61	23	18	25.1	0.26*	0.28	0.23
*Aeromonas*	BTU_111	97	37	14	21.9	0.21*	0.27	0.23
*Rickettsiella*	BTU_272	40	26	15	19.3	0.23*	0.53	0.35
*Carnobacterium*	BTU_142	142	44	12	17.4	0.29*	0.23	0.16
*Ureaplasma*	BTU_219	34	20	15	13.3	0.51*	0.52	0.40
*Arsenophonus*	BTU_137	64	32	20	13.2	0.25*	0.435	0.31
*Catellicoccus*	BTU_45	29	15	13	11.2	0.22*	0.40	0.38
*Wolbachia*	BTU_731	63	33	14	9.2	0.25*	0.40	0.26

*Note*: Details on ASVs richness and number of host's species is provided. Only unambiguously assigned BTUs exhibiting high GM‐host species specificity (*R* ^2^adj > 0.2, FDR < 0.05) or significant GM‐host co‐divergence (P_COMP_/P_PHYLO_ FDR < 0.05) are shown. GM‐host species specificity (*R* ^2^adj) and compositional GM‐host co‐divergence (P_COMP_) are based on Jaccard distances only. * indicates significant test result (FDR < 0.05). Results for all tested BTUs are provided in Table S3.

Unlike GM‐host species specificity, compositional GM‐host co‐divergence was only significant for three of all the reference‐based BTUs tested, containing namely genera *Lactococcus, Enterococcus* and *Clostridium (*sensu stricto*)* 1 (see Figure S3). Analysis of phylogenetic GM‐host co‐divergence identified three significantly co‐diverging bacterial genera, namely *Curtobacterium*, *Lactococcus*, and *Escherichia/Shigella* (FDR < 0.05; Table 1, Figure 3).

### 3.4 Reference‐free BTUs analysis

**FIGURE 5:**
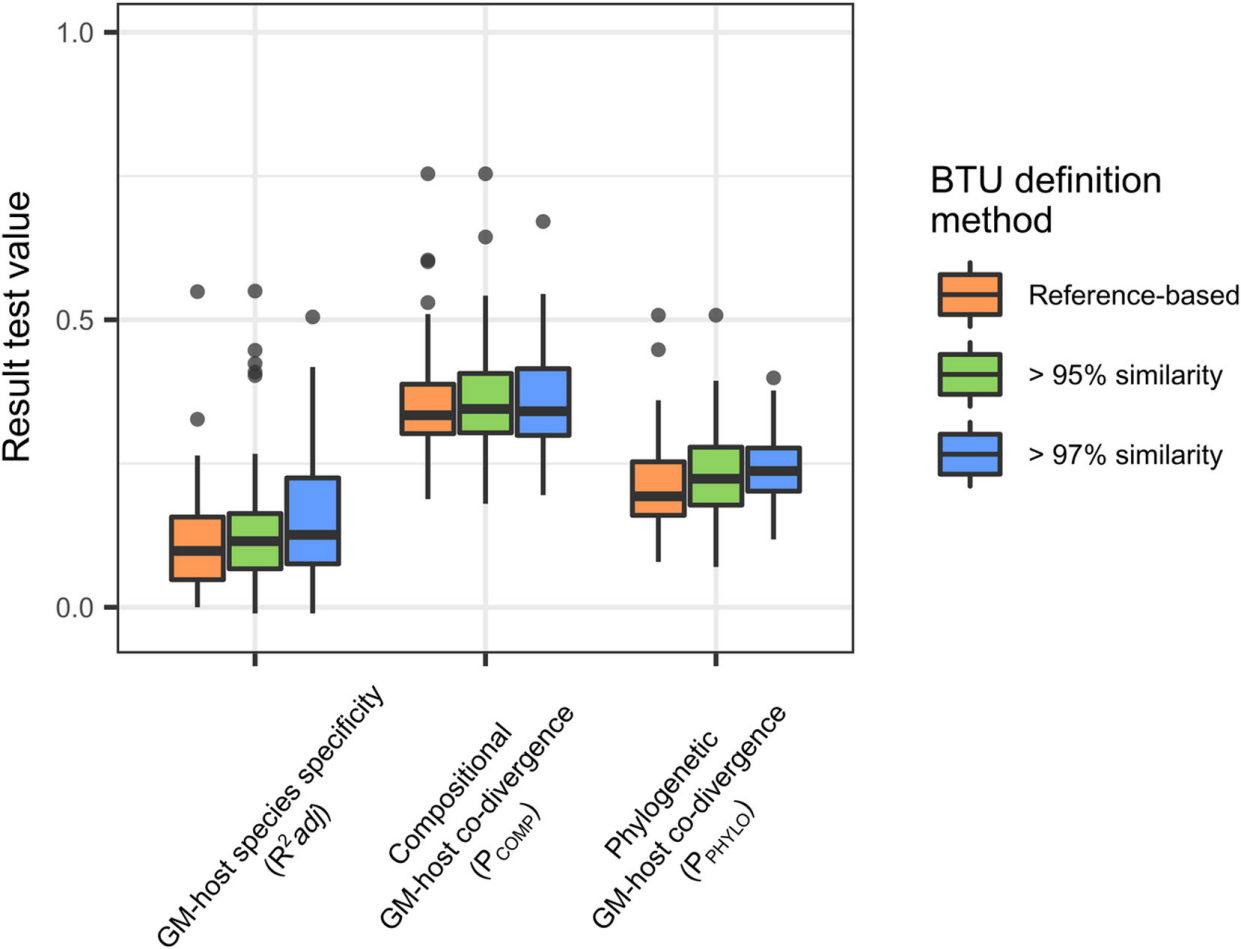
Variation in resulting effect sizes among BTU definition methods. Box‐plots showing *R* ^2^adj values in the case of GM‐host species specificity and Procrustes correlation coefficients in the case of P_COMP_ or P_PHYLO_ using different methods of BTU definition. Outliers are marked by black points

Clustering ASVs based on similarity thresholds resulted in more BTUs of lower within‐BTU ASV richness than BTUs defined by genus identity (see Table S2). The mean strength of GM‐host species specificity tended to be higher for 97% and 95% similarity‐based BTUs than for reference‐based BTUs, but the difference lacked significance (ANOVA: *F*_[2161]_ = 1.154, *p* = .318), with similar results obtained for phylogenetic GM‐host co‐divergence calculated for reference‐based versus reference‐free BTUs (ANOVA: *F*_[2161]_ = 2.370, *p* = .097; Figure 5).

As with reference‐based BTUs analysis, most similarity‐based GM BTUs provided high support (FDR <0.05) for GM‐host species specificity in ASV composition (84% of tested BTUs at the 95% similarity and 65% at the 97% similarity). However, unlike reference‐based BTUs analysis, analysis on 95% or 97% similarity‐based BTUs identified additional sets of taxa exhibiting pronounced GM‐host species specificity. These were assigned to the genera *Ureaplasma, Mycoplasma, Catellicoccus, Aeromonas, Rickettsiella*, and *Wolbachia,* along with few BTUs with ambiguous or any ASV assignment.

Finally, only two 95% similarity‐based BTUs (both ambiguously assigned on genus level) exhibited significant compositional GM‐host co‐divergence (P_COMP_ FDR <0.05) and two other 95% similarity‐based BTUs exhibited significant phylogenetic GM‐host co‐divergence (P_PHYLO_ FDR <0.05), one of these assigned exclusively to genus *Escherichia/Shigella*, similarly as for the reference‐based BTUs analysis.

## 4 DISCUSSION

In line with our previous study (Kropáčková et al., 2017), our current analysis of whole GM community versus host co‐divergence (based on all ASVs unpartitioned to BTUs) confirmed that host phylogeny acts as a significant predictor of GM variation between host species. Importantly, host's phylogeny better explained GM variation than geographic effects or host's ecological divergence (Kropáčková et al., 2017). This finding is also consistent with trends observed in studies on phylogenetically broader subsets of birds (Hird et al., 2015; Waite & Taylor, 2014). Nevertheless, despite being statistically significant, the whole GM‐host co‐divergence explained a relatively small fraction of variation between the host species included in our study. This is in agreement with comparative GM analyses showing generally weaker GM‐host co‐divergence in birds compared to other vertebrate groups and especially mammals (Song et al., 2020; Youngblut et al., 2019), likely due to flight adaptations that constrain digestive tract morphology and physiology (Bodawatta et al., 2021). Interestingly, additional analyses in (Kropáčková et al., 2017) based on an approach of Sanders et al. (2014), did not support that GM‐host co‐divergence was driven by shared evolution between hosts and their microbes mediated by stable trans‐generation GM transmission. As an alternative explanation, we proposed the co‐divergence could arise as a by‐product of host's traits not included in the analyses (e.g., immune system functions) that correlate with passerine phylogeny and shape their GM.

Here we focused on GM co‐divergence in passerine species in more detail by quantifying the strength of GM‐host co‐divergence for individual ASVs grouped within distinct BTUs. Each BTU includes phylogenetically related ASVs and thus represents more relevant entity for co‐divergence analyses than unpartitioned profiles of all ASVs (i.e., whole GM community analyses). Such a decomposition allowed us to identify individual bacterial groups exhibiting tight associations with host species. If shared evolution between GM and their hosts was a dominating factor shaping GM‐host co‐divergence, one could expect to observe strong co‐divergence signal also within individual, presumably monophyletic BTUs. However, contrary to this prediction, we showed that just a limited fraction of BTUs exhibited significant co‐divergence with the phylogeny of their hosts. Importantly this finding was robust against BTU definition method and the type of co‐divergence analysis (i.e., P_COMP_ vs. P_PHYLO_). Thus, we argue that whole GM‐host co‐divergence is unlikely to be driven by ASVs co‐divergence within individual BTUs and that host's shared evolutionary history between passerines and their GM is not the major force driving interspecific GM divergence in passerines. Therefore, the whole GM community co‐divergence is probably caused by abundance variation of unrelated ASVs that do not co‐occur in the same BTU. In a previous study by Kropáčková et al. (2017), 16 s rRNA reads were clustered into Operational Taxonomic Units assuming a range of sequence similarity thresholds (91–99%). Variation in the relative abundances of these clusters was subsequently used to analyze overall host‐GM co‐divergence. The resulting strength of the co‐divergence signal did not increase as the similarity threshold for clustering increased. This suggests, in agreement with our study, that the whole GM co‐divergence is caused by abundance changes of relatively unrelated bacteria between host species.

Also, we cannot exclude the possibility that ASVs from some low abundance BTUs (i.e. not included in BTU analysis) could have contributed to the total co‐divergence pattern.

In recent years, efforts to formalize a tight integration of GM into host biology in the evolutionary framework has resulted in the proposal of the “holobiont” concept (Zilber‐Rosenberg & Rosenberg, 2008). The original idea behind this framework (Bordenstein & Theis, 2015) assumes that the association between host and its microbiota exhibit pronounced stability over myriad of generations. This causes that host and the microbiota form a joint phenotype of evolutionary relevance where both these players undergo similar selection pressures. Shared selection can promote mutual host‐GM co‐evolution and other microevolutionary processes. However, such a radical view has also been a source of criticism (Douglas & Werren, 2016; Moran & Sloan, 2015). Our study adds further piece of empirical evidence to this skepticism by showing that divergence of ASVs within individual BTUs rarely follows phylogenetic divergence of their passerine hosts and thus that GM‐host association exhibit only a limited stability over evolutionary timescale.

Host species specificity analyses revealed that the majority of BTUs analyzed exhibited significant variation in the distribution of ASVs among host species, but this variation was mostly of moderate effect size. However, a few BTUs analyzed showed relatively high host species specificity, suggesting that there is a close association between host and specific bacterial ASVs within these bacterial clades. This could be consequence of several reasons, such as close connection of the host with sources of these bacteria (e.g., diet or habitat), or a fine‐tuned intrinsic mechanism of the host selectively regulating the population of symbiotic bacteria. Although our contribution is not directly aimed at testing these mechanisms, we believe it is useful to subject these BTUs for closer investigation for possible biological function in the host. We point out namely BTU derived from genus *Candidatus Savagella,* which is a member of the Segmented Filamentous Bacteria (SFB) group. These commensals bind specifically to the intestinal wall in many vertebrate species (Thompson et al., 2012) and play an important role in the development of a host's innate and adaptive immune system (Hedblom et al., 2018) as well as provide immune system independent protection against specific pathogens (Shi et al., 2019). SFB host‐species specificity has previously been demonstrated through experimental inoculation of germ‐free rats and mice with ileal homogenate mixtures of both donor species, resulting in colonization by SFB derived from corresponding host species (Tannock et al., 1984). In this case, therefore, strong GM‐host species specificity can be explained by host‐GM adaptation through host immune system interaction. The genus *Helicobacter*, which includes several gut pathogens, also exhibited a high level of host species specificity in this study, which is consistent with the results of previous studies (e.g., Solnick & Schauer, 2001). Some members of the genus exhibit a tight association with their hosts, which is maintained by trans‐generational transfer. This has been particularly documented in *H. pylori,* whose genomic divergence recapitulates historical differentiation and migration routes of human populations (Falush, 2003; Wirth et al., 2004). The shared evolutionary history of a host and its pathogens may also induce a high host‐specific signal in other genera, including potentially pathogenic species such as *Brachyspira*, *Diplorickettsia*, *Yersinia*, *Rickettsiella, Ureaplasma, Mycoplasma, Clostridium* (sensu stricto), and *Escherichia/Shigella*. Genus *Carnobacterium*, which showed a strong host‐specific signal in our BTU analysis, produces a number of bacteriocins that inhibit the growth of potential competitors, including pathogenic *Listeria* (Pilchová et al., 2016). Such a feature could be favored by the host as it contributes to the maintenance of GM‐community homeostasis. Another group of reference‐based BTUs exhibiting high host‐specific signals, which included the cellulase‐positive genus *Cellulomonas* and a number of lactic acid bacteria (*Enterococcus*, *Lactococcus*, and *Catellicoccus*), was characterized by complex carbohydrate utilization, giving them the potential to provide additional nutrients from compounds not degradable by the host (Jami et al., 2013). In the case of *Lactococcus* and *Enterococcus*, ASV distribution was also correlated with host species phylogeny. A very high level of host species specificity has previously been demonstrated for *Lactococcus* in humans, other various mammals and poultry (Santagati et al., 2012). In addition to the above mentioned groups, *Wolbachia* and *Arsenophonus*, which include obligatory insect symbionts (Gherna et al., 1991; Sharon et al., 2010) were also unevenly distributed among the passerine species sampled, possibly as a consequence of interspecific differences in diet composition.

In conclusion, we have shown that using individual BTUs to analyze various aspects of GM variability (host species specificity and co‐divergence with host phylogeny in our particular case) can provide valuable insights that cannot be achieved with traditional whole‐GM community approaches. In particular, based on low host versus GM co‐divergence at the BTU level, we propose that a shared phylogenetic history between the host and its GM is not the major force driving passerine GM diversity or co‐divergence between host phylogeny and GM composition. This represents a potentially valuable contribution to the recent debate on the nature of coexistence of bacterial communities associated with vertebrate host (Bordenstein & Theis, 2015; Douglas & Werren, 2016; Moran & Sloan, 2015; Rosenberg & Zilber‐Rosenberg, 2011). At the same time, however species specificity at the BTU level was commonly detected in our dataset. Based on the features of the reference‐based BTUs showing high level of host species specificity, our results suggest the existence of a range of mechanisms contributing to this variation. At present, however, the proposed explanations for these mechanisms are based on putative interactions that require further investigation.

## AUTHOR CONTRIBUTIONS

**Jan Kubovčiak:** Conceptualization (equal); formal analysis (lead); funding acquisition (equal); investigation (equal); visualization (lead); writing – original draft (equal); writing – review and editing (equal). **Lucie Schmiedova:** Formal analysis (equal); methodology (equal); writing – review and editing (supporting). **Tomas Albrecht:** Resources (equal). **Martin Těšický:** Resources (equal). **Oldřich Tomášek:** Resources (equal). **Tereza Kauzálová:** Resources (equal). **Jakub Kreisinger:** Conceptualization (equal); data curation (lead); funding acquisition (equal); investigation (equal); methodology (equal); supervision (lead); writing – original draft (equal); writing – review and editing (supporting).

## CONFLICT OF INTEREST

All authors declare no conflict of interest.

## Supporting information


Figure S1

Figure S2

Figure S3

Table S1

Table S2

Table S3

Table S4

## Data Availability

The sequencing data are archived in the European Nucleotide Archive under the accession number of the entire project PRJEB53462.
