# Supplementary figures and images for: Within‐community variation of interspecific divergence patterns in passerine gut microbiota
Source: Ecol Evol. 2022 Jul 4;12(7):e9071. doi: 10.1002/ece3.9071 (PMC9251858; doi:10.1002/ece3.9071)

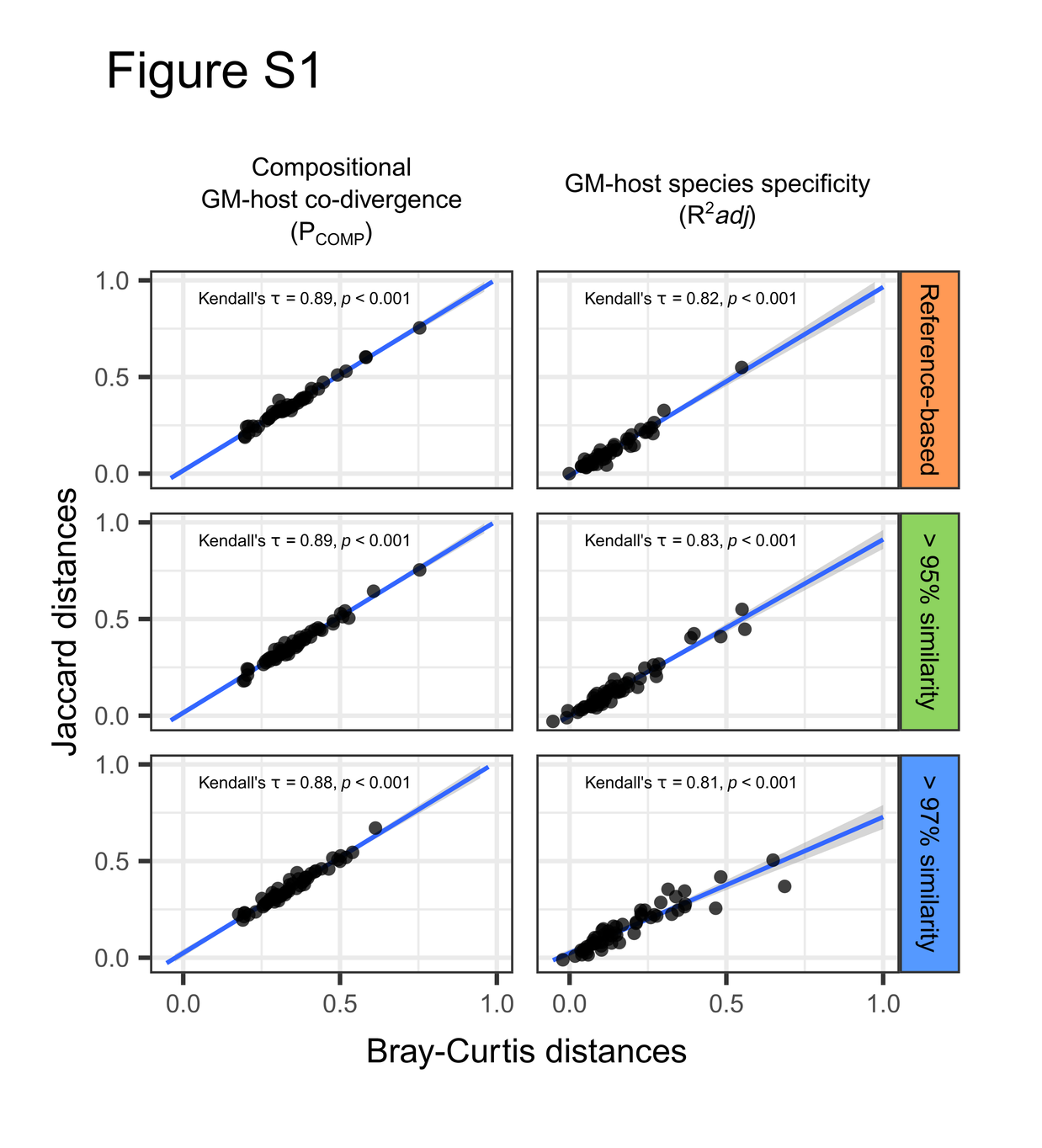

Supplement: Supplementary file 1 — Figure S1 [file ECE3-12-e9071-s005.tif]

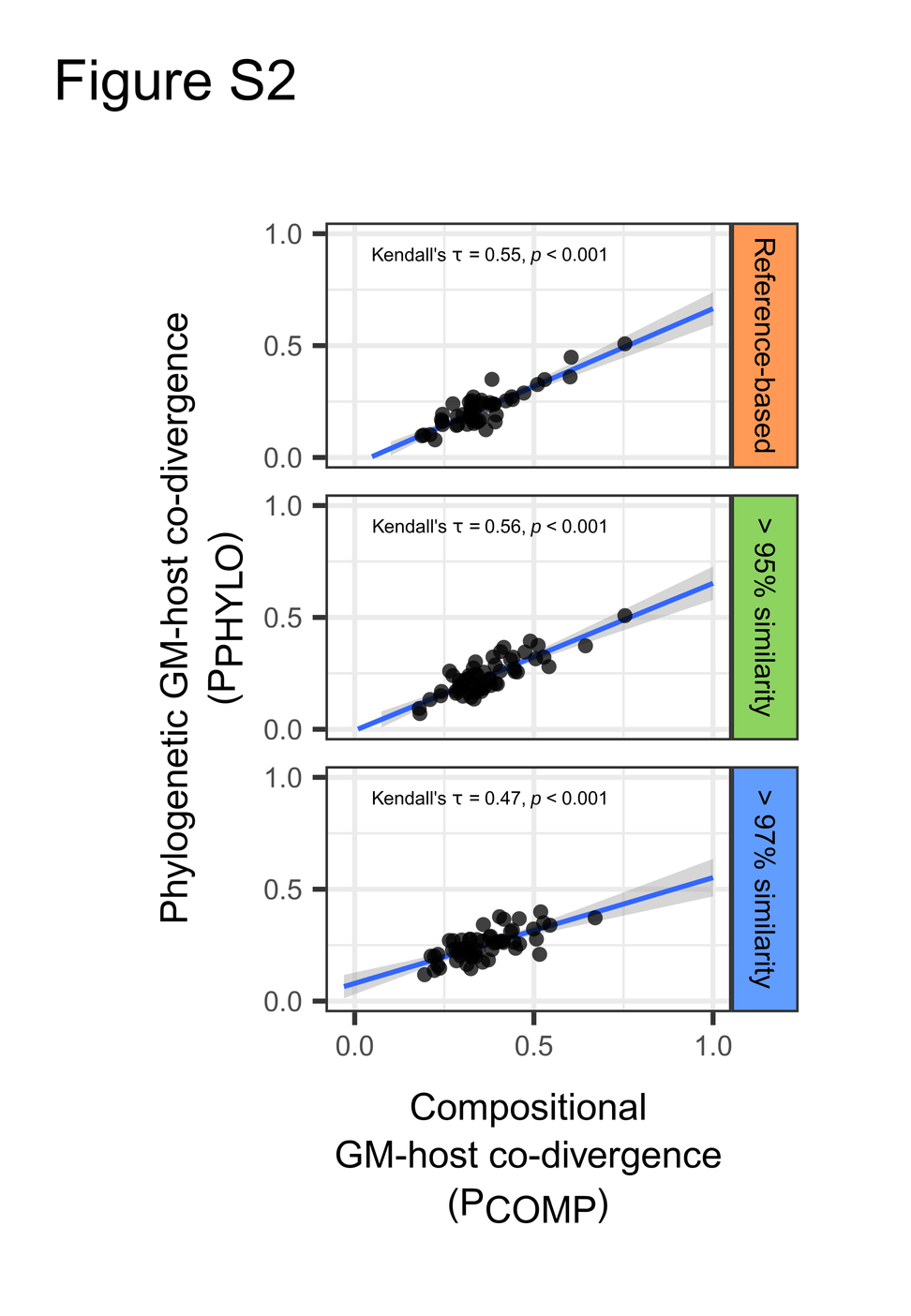

Supplement: Supplementary file 2 — Figure S2 [file ECE3-12-e9071-s004.tif]

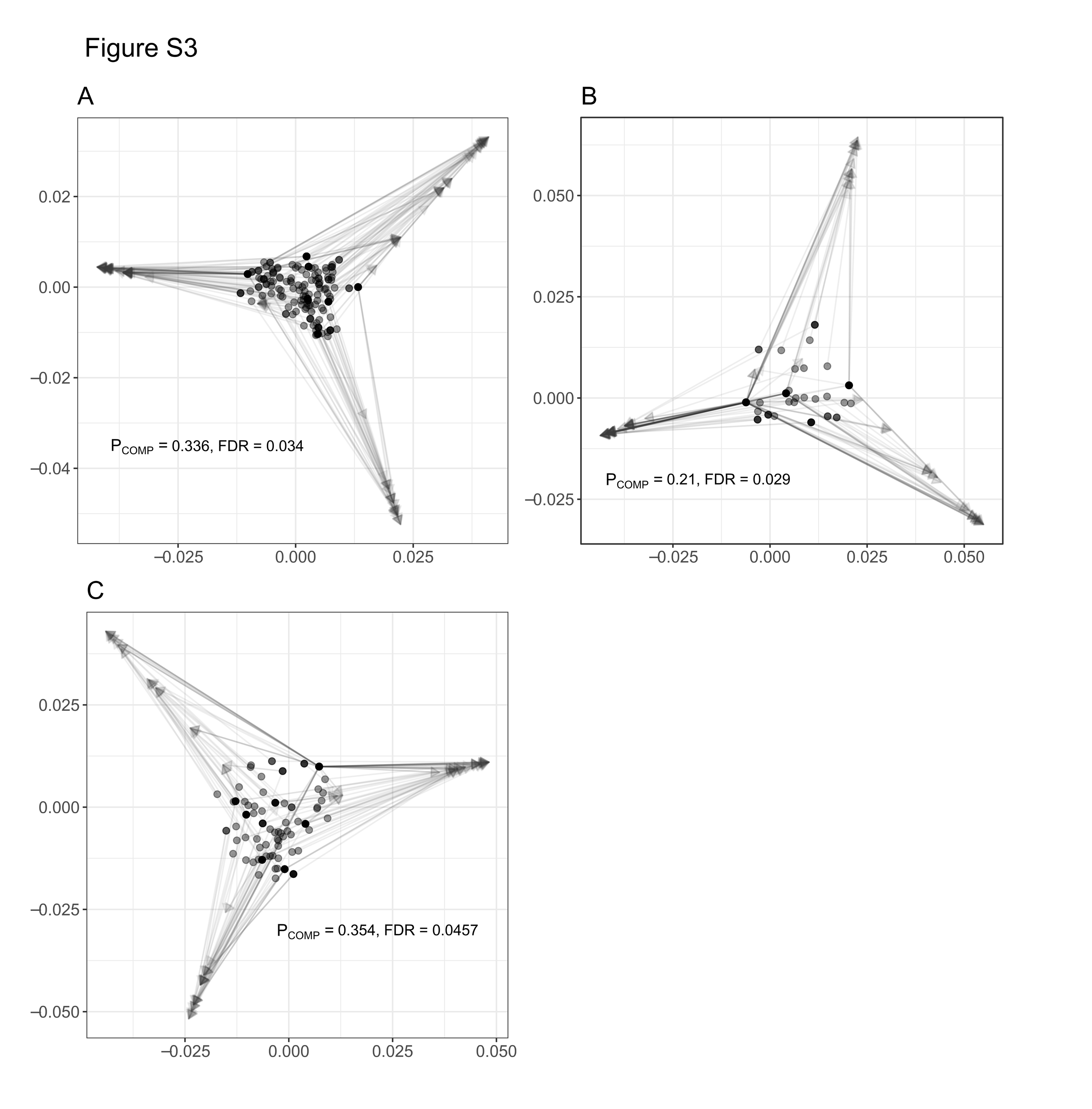

Supplement: Supplementary file 3 — Figure S3 [file ECE3-12-e9071-s001.tif]
